# Genome sequences of four agarolytic bacteria from the Bacteroidia and Gammaproteobacteria

**DOI:** 10.1128/MRA.00667-23

**Published:** 2023-10-09

**Authors:** Elise K. Phillips, Jacob M. C. Shaffer, Michael W. Henson, Jordan T. Coelho, Mark O. Martin, J. Cameron Thrash

**Affiliations:** 1 Department of Biology, University of Puget Sound, Tacoma, Washington, USA; 2 Department of Geophysical Sciences, University of Chicago, Chicago, Illinois, USA; 3 Department of Biological Sciences, University of Southern California, Los Angeles, California, USA; Montana State University, Bozeman, Montana, USA

**Keywords:** agarolytic, agarase, marine microbiology

## Abstract

Here we present the genomes of four marine agarolytic bacteria belonging to the Bacteroidota and Proteobacteria. Two genomes are closed and two are in draft form, but all are at least 99% complete and offer new opportunities to study agar-degradation in marine bacteria.

## ANNOUNCEMENT

Microorganisms capable of degrading agar (agarolytic) are important for understanding carbon cycling in marine systems, interactions with agar-producing organisms, and human digestion, and have various biotechnological applications (1 – 7). Agar degradation is a widespread phenotype, conferred by multiple different agarases in the CAZyme glycoside hydrolase family with unique agar bond specificities that are deployed in a variety of combinations depending on the organism (3, 8 – 11). Here, we present the sequenced genomes of four bacteria with phenotypic and genotypic evidence of agarolytic activity isolated from multiple marine sources to expand and explore the genomics underlying this metabolism.

**Fig 1:**
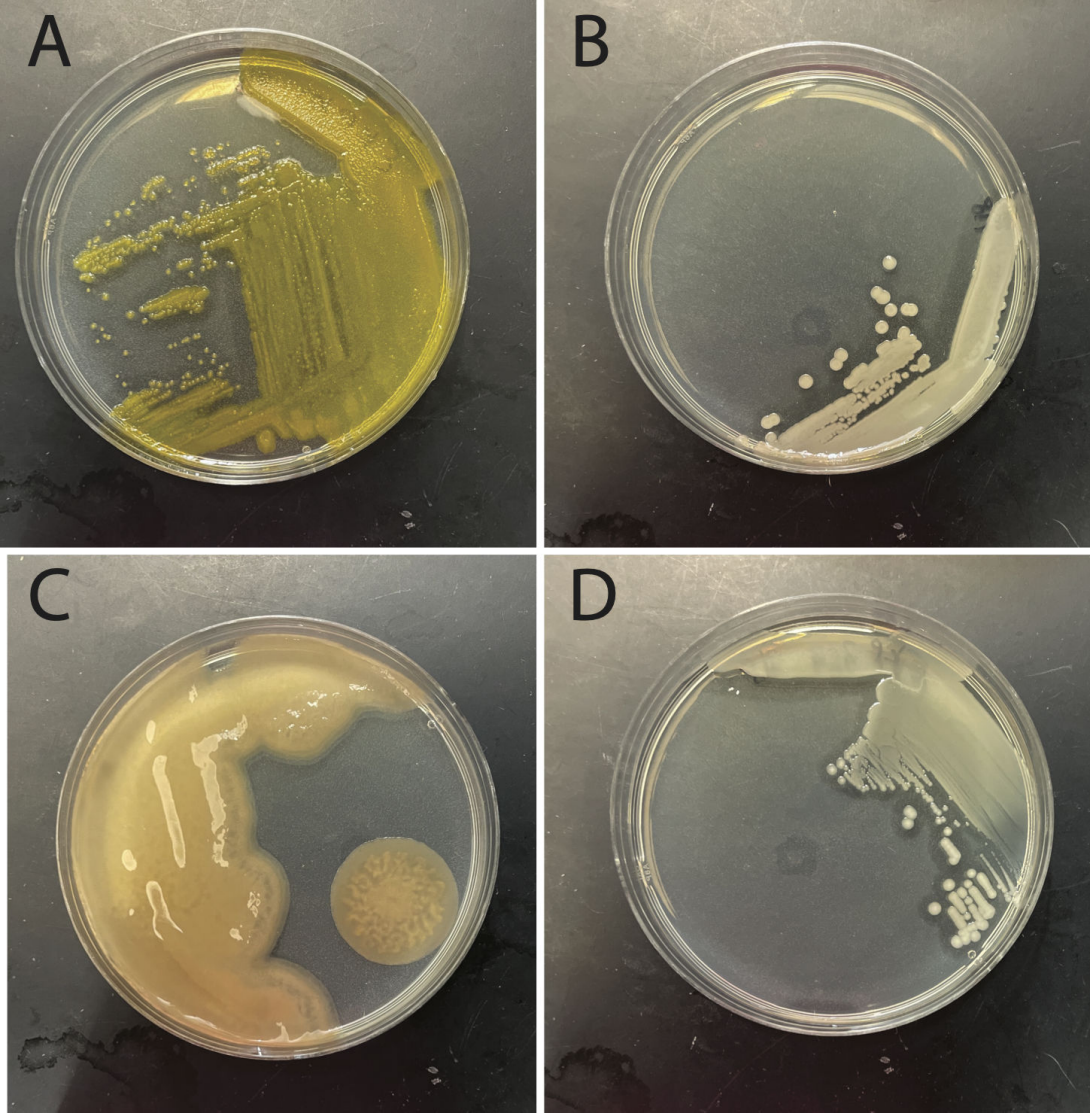
Colony morphology and phenotypes of the four strains. (**A**) *Cellulophaga omnivescoria* strain EKP101, (**B**) *Vibrio* sp. strain EKP203, (**C**) *Flammeovirga* sp. strain EKP202, and (**D**) *Pseudoalteromonas distincta* strain EKP108. The zones of clearing around the colonies indicate areas of agarolytic activity.

We collected surface seawater samples (15 mL) in Falcon tubes from beaches in Washington State and Key West, Florida (Table 1), transported these to the Martin Lab at the University of Puget Sound on ice, and stored them at 12°C until plating shortly thereafter. We serially diluted samples using artificial seawater before plating 0.1 mL on seawater complete (SWC) agar plates (12). We identified colonies with conspicuous agarolytic “pitting” phenotypes (Fig. 1) and passaged organisms of interest twice to ensure isolation. For generating frozen stocks and extracting genomic DNA, we inoculated cultures in SWC broth with 0.1% agar, which we incubated overnight in a shaking water bath (30°C). Cryostocks included 25% sterile glycerol and were stored at −80°C. High molecular weight DNA was extracted using a PureLink Microbiome DNA Extraction Kit (Invitrogen, USA), purified using a DNA Clean and Concentrator Kit (Zymo Research, USA), and quantified via Qubit Fluorometer (Invitrogen, USA).

**TABLE 1:** Genome summary statistics, taxonomy, and isolation information^*a*^

	NCBI				Quast v5.2.0					CoverM v0.4.0				
Strain	Genome accession	SRA accession Illumina	SRA accession Nanopore	BioSample	Genome size	N50	GC content	Illumina reads	Nanopore reads	Avg cov Illumina	Avg cov Nanopore	Contigs	Circularized	Extrachromosomal elements
EKP101	CP129325	SRX9092153	SRX9092157	SAMN16076811	3873362	3873362	32.1	11,812,658	7,669	447.2	28.7	1	Yes	No
EKP203	JAUEOZ000000000	SRX9092156	SRX9092158	SAMN16076814	4784174	3004313	45.4	11,648,610	9,791	356.3	22.4	3	Yes	Yes (1,599,951 bp; 179,910 bp)
EKP202	JACVVP000000000	SRX9092155	NA	SAMN16076813	7997971	126188	34.8	14,523,450	NA	265.3	NA	183	No	Unknown
EKP108	JACVVQ000000000	SRX9092154	NA	SAMN16076812	4525022	146029	39.2	12,868,864	NA	414.5	NA	73	No	Unknown

	GTDB-tk v2.1.1			CheckM2 v1.0.0								
	user_genome	Classification	species ANI	Compl^*b*^	Cont^*b*^	CD^*b*^	Summary nomenclature	Shaffer Thesis nomenclature	Isolation site	Isolation date	Predicted agarases	Phenotype
EKP101	Jacob1_112519_assebmly	d__Bacteria;p__Bacteroidota;c__Bacteroidia;o__Flavobacteriales;f__Flavobacteriaceae;g__Cellulophaga;s__Cellulophaga omnivescoria	99.07	100	0.05	0.915	Jacob1	DPYP - Dash Point Yellow Pitter	Dash Point Beach, Washington (47.3203° N, 122.4142° W)	Fall 2017	Beta-agarase x2	Small yellow colonies, shallow agar pitting.
EKP203	Jacob3_112519_assembly	d__Bacteria;p__Proteobacteria;c__Gammaproteobacteria;o__Enterobacterales;f__Vibrionaceae;g__Vibrio;s__	NA	100	0.62	0.868	Jacob3	KWSWP - Key West Small White Pitter	Key West, Florida (24°32’49.54”N, 81°47’31.25”W)	Spring 2018	Beta-agarase x1	Small white colonies, deep agar pitting.
EKP202	Jacob4_contigs_SPades	d__Bacteria;p__Bacteroidota;c__Bacteroidia;o__Cytophagales;f__Flammeovirgaceae;g__Flammeovirga;s__Flammeovirga sp014534745	NA	99.98	1.37	0.864	Jacob4	KWMM - Key West Mango Migrator	Key West, Florida (24°32’49.54”N, 81°47’31.25”W)	Spring 2018	Beta-agarase x1	Rapidly growing orange film, shallow wide pitting of agar (dissolves agar underneath spreading growth).
EKP108	Jacob5_contigs_SPades	d__Bacteria;p__Proteobacteria;c__Gammaproteobacteria;o__Enterobacterales;f__Alteromonadaceae;g__Pseudoalteromonas;s__Pseudoalteromonas distincta	97.16	100	0.11	0.882	Jacob5	OWP - Owens Beach Pitter	Owen Beach, Washington (47.3134° N, 122.5287° W)	Spring 2018	Agarase x1	Large white colonies, deep and wide agar pitting.

^*a*^ NA, not applicable.

^*b*^ Compl, completion; Cont, contamination; CD, coding density.

We used Illumina sequencing for all strains and additional Oxford Nanopore sequencing for two. The same DNA extractions were used for Nanopore and Illumina sequencing. We sheared DNA for Nanopore sequencing to 4–8 kbp with a G-tube (Covaris) at 6,000 rpm, constructed libraries with the SQK-LSK108 1D genomic DNA ligation kit (Oxford Nanopore, UK) with slight modifications (https://doi.org/10.17504/protocols.io.bixskfne), and sequenced with an R9.4 flowcell using a MinION (Oxford Nanopore Technologies, ONT). Base-calling was performed with Guppy v2.3.1 (ONT). Illumina library preparation and sequencing were performed as previously described (13) at the USC Genome Core with NextSeq550 paired end 150 bp sequencing. Hybrid Nanopore-Illumina assemblies for strains EKP101 and EKP203 were performed with Unicycler v0.4.8 (14), and Illumina-only assemblies were performed for strains EKP108 and EKP202 with SPAdes v3.13.1 (15). We performed post-assembly assessment with CheckM2 v1.0.0 *predict* (16), Quast v5.0.2 (17), coverage calculations with CoverM 0.4.0 (https://github.com/wwood/CoverM), and taxonomic identification with GTDB-tk v2.1.1 *classify_wf* (18). Default settings were used for all software unless otherwise noted.

Sequencing and assembly results are in Table 1. The taxonomy is as follows: *Cellulophaga omnivescoria* strain EKP101; *Flammeovirga* sp. strain EKP202 (Class Bacteroidia); *Vibrio* sp. strain EKP203; and *Pseudoalteromonas distincta* strain EKP108 (Class Gammaproteobacteria). The two hybrid assemblies (EKP101 and EKP203) are circularized, and *Vibrio* sp. strain EKP203 contains two chromosomes and a putative plasmid based on all three replicons being circularized at 3,004,313 bp, 1,599,951 bp, and 179,910 bp (Table 1). All assemblies were estimated at greater than 99% complete with minimal estimated redundancy/contamination and contained predicted agarases (Table 1). Physiology and preliminary analysis of predicted agarase genes were completed in the Honors Theses of E. Philips and J. Shaffer, respectively (dois: 10.6084/m9.figshare.23727165; 10.6084/m9.figshare.23727168   ).

## Data Availability

Genome sequences and raw sequencing reads are available under BioProject number PRJNA662106. Individual genome and SRA accessions are provided in Table 1.
